# Triptolide: A Narrative Review of Its Traditional Use, Derivatives, Pharmacology, Antitumor Effect, and Clinical Applications

**DOI:** 10.3390/cancers18081196

**Published:** 2026-04-09

**Authors:** Yibo Geng, Bettina Kritzer, Javad Nazarian

**Affiliations:** 1 DIPG/DMG Center Zurich, Children’s Research Center, University Children’s Hospital Zurich, University of Zurich, August-Forel-Strasse 51, 8008 Zurich, Switzerland; yibo.geng@kispi.uzh.ch (Y.G.); bettina.kritzer@kispi.uzh.ch (B.K.); 2 Department of Neurosurgry, Beijing Chaoyang Hospital, Capital Medical University, No. 8 Gongti South Road, Beijing 100020, China

**Keywords:** triptolide, Minnelide, prodrugs, *Tripterygium wilfordii*, TPL

## Abstract

Triptolide (TPL), a diterpenoid derived from the Chinese medicinal plant *Tripterygium wilfordii*, exhibits broad-spectrum biological and pharmacological activities, although its clinical translation is limited by systemic toxicity. Recent advances in the development of TPL derivatives have created new therapeutic opportunities. This review summarizes current knowledge of triptolide, with a focus on TPL’s toxicity profile, derivative strategies, and antitumor mechanisms across different tumor types, including glioma, pancreatic tumor, leukemia, lung cancer, gastric cancer and others. We also summarize the plant’s origin and traditional uses, TPL’s pharmacokinetics (PKs), and relevant clinical trials against tumors. The main mechanism of the TPL antitumor effect is to interfere with ATPase of XPB by covalently binding to it, as well as inducing the rapid depletion of RPB1 via hyperphosphorylation and ubiquitination. We also reviewed systemic toxicity including neuro-, cardio-, oto-, nephron-, hepato-, and hemato-toxicity, as well as digestive and reproductive toxicity. Finally, we searched clinical trial databases across three platforms for tumors and concluded that Minnelide has strong clinical potential for solid tumors. By critically evaluating TPL from multiple dimensions, specifically its traditional use, chemical derivatization, pharmacokinetics, antitumor mechanisms, toxicity, and clinical trials, this review aims to inform future strategies that maximize therapeutic efficacy while minimizing adverse effects.

## Simple Summary

Triptolide, a compound from a traditional medicinal plant, shows strong anticancer effects but with observed toxicities. As such, its clinical utility may be limited. New derivatives are being developed to overcome this problem. This review summarises the natural source of triptolide and its traditional uses. We also highlight drug metabolism and its mechanisms of action in killing tumor cells. We further discuss prodrugs and their efficacy and potential toxicities. We then discuss registered clinical trials of promising derivative called Minnelide. By evaluating both the potential and the risks of its use, this review aims to guide the development of safer, more effective triptolide-based cancer therapies.

## 1. Introduction

*Tripterygium wilfordii Hook. f. (TwHF)*, known in Chinese as “Leigongteng”, holds a significant place in traditional Chinese medicine, with documented uses dating back centuries [1]. Despite its historical application in treating conditions including rheumatoid arthritis and skin disorders, the TPL’s apparent toxicity has limited its wider clinical applications. Triptolide (TPL), a key bioactive diterpenoid triepoxide, was isolated from *TwHF* in the 1970s and was found to have pharmacological potential, particularly as a potent antitumor agent in medulloblastoma, pancreatic cancer, and leukemia in vivo [2,3,4]. The TPL mechanism of action includes indirect degradation of RNA polymerase II and inhibition of the transcription factor XPB [5]. As such, TPL has shown efficacy across a wide spectrum of cancers, including glioma, pancreatic cancer, lung cancer, and leukemia. However, the clinical translation of TPL has been hampered by its poor solubility and systemic toxicity [6]. In recent years, extensive research has focused on overcoming these challenges through the synthesis of novel derivatives and the development of advanced drug-delivery systems [7].

Recently, several reviews have focused on distinct aspects of TPL. Cui et al. reviewed 12 anticancer compounds derived from herbal medicine and summarized the roles of TPL in the tumor immune microenvironment, drug resistance, mechanisms, toxicity and prodrug development across various tumor types [8]. Feng et al. provided a comprehensive overview centered on the anticancer mechanism and derivatives of TPL [9]. AbdulHussein et al. focused on the mechanisms of cell death induced by TPL in various cancers, including apoptosis, autophagy, senescence, pyroptosis and necrosis [10]. In addition, several reviews have concentrated on specific organs or systems, such as gynecological, hematological, breast, liver, lung and pancreatic cancer [7,11,12,13,14]. Although these reviews synthesized original research and provided valuable insight into their respective topics, most lack illustrative figures or tables, which may hinder readers’ understanding. Thus, this review aims to provide a comprehensive, reader-friendly and up-to-date synthesis of the traditional uses, chemical derivatization, pharmacology, antitumor mechanisms, and the current status of clinical trials of TPL and its derivatives. By critically evaluating the gap between TPL’s antitumor ability and clinical utility, we highlight both promising therapeutic avenues and persistent challenges in its development as a modern oncotherapeutic drug.

## 2. Plant *TwHF* Characteristics and Geographic Distribution

The plant *TwHF* (also known as Thunder God Vine) is a traditional Chinese botanical medicine, first documented in the Compendium of Materia Medica in the 16th century [1]. The plant’s Chinese name (Leigongteng) alludes to the plant’s potent toxicity. The plant is cultivated across Asia, including Korea, Japan, and India. *TwHF* was introduced to the United States in the 1930s and has since attracted research interest from scientists worldwide. This plant is a deciduous subshrub or a climbing semi-woody vine, and its root and rhizomes are highly important in traditional Chinese medicine. *TwHF* belongs to the genus *Tripterygium* (*Celastraceae*), which includes several closely related species, such as *T. hypoglaucum* and *T. regelii* [15], which are also toxic and exhibit similar antitumor and immunomodulatory effects.

## 3. Traditional Uses of *TwHF* and Chemical Structure of TPL

The traditional uses of *TwHF* were documented in Chinese medical literature as early as the Ming Dynasty. No non-Chinese medicinal use of *TwHF* was documented prior to modern times, likely due to its restricted geographical distribution. Given its significant toxicity, *TwHF* was traditionally applied topically rather than orally. A summary of its traditional uses is provided in Table 1.

**Figure 1:**
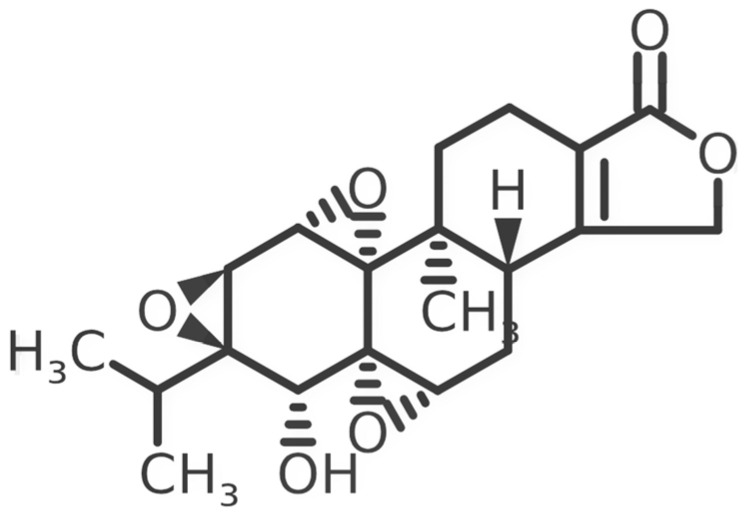
Chemical structure of TPL. (Adopted from FDA website https://precision.fda.gov/home, accessed on 19 January 2026).

In 1972, a compound called TPL was extracted from the roots of the *TwHF* plant [16]. X-ray crystallography showed that TPL consists of a 5/7/5 tricyclic diterpenoid canonical skeleton, a highly strained and reactive three-membered epoxy bridge (C-12/C-13), and a α,β-unsaturated five-membered lactone ring (Figure 1).

**Table 1:** Traditional uses of *TwHF*.

**The Name or Origin of Traditional Uses**	**Main Components**	Usage	Reference
*TwHF*	Leaves of *TWHF*	Itchy skin, external use	https://www.hnysfww.com/mobile/goods.php?id=9024 (accessed on 12 November 2025)
*TwHF*	Flower of *TWHF*, *Lindera aggregata* (*Sims*) *Kosterm*	Skin sores, external use	https://www.hnysfww.com/mobile/goods.php?id=9024 (accessed on 12 November 2025)
HUO BA HUA GEN Tablet	Root of *T. hypoglaucum*	Psoriasis, chronic nephritis	https://d.wanfangdata.com.cn/periodical/CiBQZXJpb2RpY2FsQ0hJU29scjkyMDI2MDMwNjE2NTI1NxIUemd6eHlqaHNienoyMDAzMDcwMTgaCGxiMmY4enkz (accessed on 12 November 2025) https://d.wanfangdata.com.cn/periodical/CiBQZXJpb2RpY2FsQ0hJU29scjkyMDI2MDMwNjE2NTI1NxINemhwZjIwMDAwMjAzMhoIbHdxZ3g0bDQ%3D (accessed on 12 November 2025)
Tripterygium glycosides tablets	Tripterygium glycosides	Rheumatoid arthritis, nephrotic syndrome,	https://d.wanfangdata.com.cn/periodical/CiBQZXJpb2RpY2FsQ0hJU29scjkyMDI2MDMwNjE2NTI1NxIUZ3VhbmdkeXh5eGIyMDAyMDMwMzYaCHN4OXhleXdq (accessed on 12 November 2025)
*TwHF* tablets	Triptolide	Rheumatoid arthritis, nephrotic syndrome,	https://d.wanfangdata.com.cn/thesis/Ch1UaGVzaXNOZXdTb2xyOVMyMDI2MDExNzA4NTkxNhIHWTgwMzUzNRoIZDZkZmNuc3g%3D (accessed on 12 November 2025) https://d.wanfangdata.com.cn/periodical/CiBQZXJpb2RpY2FsQ0hJU29scjkyMDI2MDMwNjE2NTI1NxINdGp6eTIwMjEwMjAxNhoINTR6aDV0d24%3D (accessed on 12 November 2025)
Jin Guan Tablets	*TwHF*, *Dipsacus asper Wall. ex Henry*, *Chinese yam*, *Asarum heterotropoides F. Schmidt*, etc.	Rheumatoid arthritis, ankylosing spondylitis	https://d.wanfangdata.com.cn/periodical/CiBQZXJpb2RpY2FsQ0hJU29scjkyMDI2MDMwNjE2NTI1NxIOenh5amgyMDA5MDIwMDIaCGtvcWpjemNy (accessed on 12 November 2025)
Shuangniu Trauma Medicinal Wine	Root of *TwHF*, *aconitum kusnezoffii*, *safflower*, etc.	Traumatic injuries, external	http://m.zhongyoo.com/yaojiu/5114.html (accessed on 12 November 2025)
Kidney disease prescription	*TwHF*, *Chrysanthemum*, *dandelion*, *bittercress*	Nephrotic syndrome	[17]
Anti-rheumatic wine	*TwHF*, *Clematis chinensis*, *Rehmannia* root, *Polygonatum sibiricum*	Rheumatoid arthritis	https://d.wanfangdata.com.cn/periodical/CiBQZXJpb2RpY2FsQ0hJU29scjkyMDI2MDMwNjE2NTI1NxINaGJ6eTIwMDMwMzAwMxoIOG95bDVjOXQ%3D (accessed on 12 November 2025)
God’s Response All-Effective Ointment	*TwHF*, *Aconitum kusnezoffii*, *Linderae sibiricum*, *Areca catechu*	All wind-induced swelling and toxic diseases	http://ethnobotany.cn/ (accessed on 12 November 2025)
Insecticide	*TwHF*	Insecticides to larva	[18]

## 4. Pharmacology

### 4.1. Toxicity

**Figure 2:**
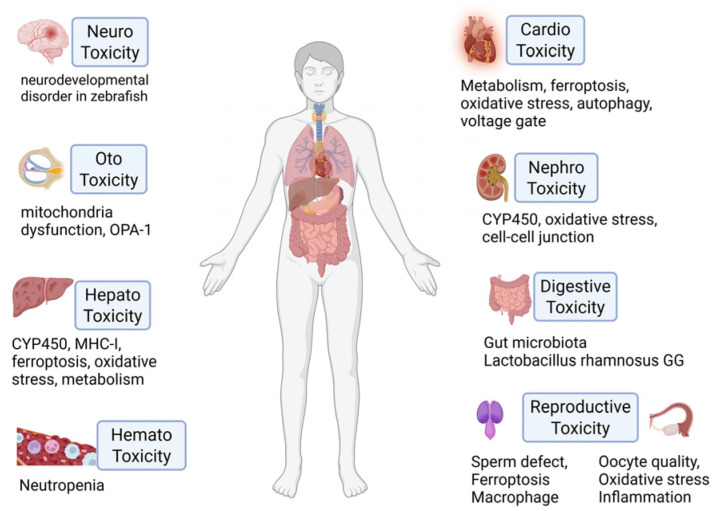
Reported systemic toxicities associated with TPL (created with BioRender.com).

The clinical application of TPL is limited by dose-limiting toxicities affecting the liver, kidneys, heart, reproductive system, and others (Figure 2). As the primary site of drug metabolism, the liver is particularly susceptible to toxicity from drugs and their metabolites. TPL-induced hepatotoxicity involves multiple intracellular signaling pathways, including the regulation of cytochrome P450 enzymes, immune cell responses, and gut microbiota imbalance. Specifically, TPL has been shown to cause hepatotoxicity by reducing the substrate affinity, activity, and expression (at both transcriptional and protein levels) of CYP450 isoforms, including 3A, 2C9, 2C19, and 2E1 [19,20,21]. Furthermore, TPL sensitizes hepatocytes to exogenous NK-cell-mediated cytotoxicity by inhibiting hepatocyte MHC-I expression [22]. Additionally, TPL-induced liver injury has been linked to Th2 cytokines produced by iNKT cells, which promote the expression of immunoregulatory factors [23]. TPL triggers iron accumulation and lipid peroxidation by modulating Nrf2 expression [24]. Interestingly, TPL perturbs the gut microbiota-bile acid-FXR axis, wherein a reduction in Lactobacillus rhamnosus GG abundance ultimately promotes liver damage [25].

The kidney is also highly vulnerable to TPL toxicity. Shen et al. demonstrated that the organic cation transporter 2, expressed on the surface of renal tubular epithelial cells, mediates TPL transfer from the blood to the renal tubule [26]. Within the tubule, TPL disrupts cell–cell junctions and increases paracellular permeability [27]. The principal mechanisms underlying TPL-induced nephrotoxicity are oxidative stress and inflammation, both of which are dose-dependent [28]. Multi-omics analyses have revealed changes in RNA and protein profiles, implicating several pathways in TPL nephrotoxicity. These include the cytochrome P450 protein family, cellular lipolytic activity, and antioxidant nuclear transcription factors, operating through acute-phase response signaling, the antigen presentation pathway, FXR/RXR activation, LPS/IL-1-mediated inhibition of RXR function, and EIF2 signaling [29,30]. Furthermore, an in vivo study confirmed that oxidative stress-induced mitochondrial DNA damage activates the cGAS-STING pathway, which leads to nephrotoxicity [28].

Research on TPL-induced cardiotoxicity emerged in the 2010s. A predominant finding is that metabolic dysregulation, implicated in approximately half of the related studies, impairs glucose uptake and glycogen metabolism [31,32]. This dysregulation promotes the generation of reactive oxygen species (ROS), inducing oxidative stress that damages cardiac mitochondria, proteins, and DNA through multiple pathways [33,34], ultimately leading to cardiomyocyte apoptosis and F-actin depolymerization [35]. Mechanistic studies have highlighted several key processes. The mitochondria-targeted antioxidant MitoQ was shown to alleviate TPL-induced cardiotoxicity by restoring NRF2 expression [36]. Similarly, calycosin, a compound known to regulate mitochondrial respiration via PGC-1α activation [37,38], protected against TPL-induced impairment of PGC-1α/NRF1-dependent mitochondrial biogenesis and respiration [39], suggesting a potential combination strategy to mitigate toxicity. Furthermore, Xu et al. used patch-clamp experiments to show that TPL binds to and inhibits the voltage-gated sodium channels Nav1.5 and Nav1.7, contributing to its cardiotoxic effects [40]. Additional reported mechanisms include SLC7A11/GPX4 inactivation-mediated ferroptosis and dysregulated autophagy [34,41,42].

TPL systemic toxicity has been studied in several preclinical in vivo models. In zebrafish embryos, TPL exposure caused a concentration-dependent reduction in mean swimming distance, suggesting neurobehavioral toxicity [43]. Paradoxically, other studies have reported neuroprotective effects in models of neurodegenerative diseases, attributed to its anti-inflammatory action on microglia [44,45]. In males, TPL caused significant testicular damage and impaired spermatogenesis. Reduced sperm concentration and aberrant morphology were evident. These effects were mediated by elevated ROS and malondialdehyde production, along with decreased glutathione levels and glutathione peroxidase 4 (GPX4) expression [46]. In females, TPL exposure diminished ovarian function and fertility, an effect driven by mitochondrial DNA release and subsequent activation of the cGAS-STING pathway [47]. Additionally, TPL administration perturbed the gut microbiota composition, notably reducing the abundance of Lactobacillus rhamnosus GG [25]. TPL also induced mitochondrial dysfunction and ROS production, causing systemic inflammatory responses in the kidney and liver [48], as well as in inner ear stem cells [49].

### 4.2. PK

A comprehensive review published in 2019 summarized the PK profile of TPL [50]. Briefly, TPL is rapidly and extensively absorbed after oral administration, exhibiting a bioavailability of approximately 75% in dogs and reaching peak plasma concentration (Tmax) within 10 min. Following oral dosing, TPL distributes extensively into major organs, including the liver, heart, spleen, lung, and kidney. TPL is metabolized primarily by human CYP2C19 and CYP3A4 enzymes, with less than 4% of the administered dose recovered unchanged in feces, bile, and urine within 24 h. It is eliminated rapidly, with reported terminal half-lives of 0.42 h (oral) and 0.19 h (intravenous) in rats. Notably, nearly 39% of the parent drug is cleared via biliary excretion post-absorption [50]. In 2020, Zhu et al. developed a strategy for synchronous measuring of TPL both in blood and brain based on mass spectrometry [51]. They found the Tmax to be 55.0 ± 12.3 min and Cmax of 15.1 ± 5.3 ng/mL after oral gavage 0.5 mg/kg for normal rats, and the area under the curve increased to 1.5-fold in rat models of Alzheimer’s disease.

Subsequent studies include TPL co-administration with paeoniflorin, the main active component of *Paeonia lactiflora*, which has anti-inflammatory properties, resulting in reduced peak concentration (Cmax) and delayed Tmax of TPL [52]. The uptake and efflux of TPL in the rat duodenum were shown to be mediated by Oatp1a5 and P-glycoprotein, respectively [53]. Pretreatment with antibiotics increased the Cmax and relative bioavailability of TPL by approximately 50%, attributable to an elevated inflammatory response [54].

### 4.3. Molecular Docking

Central to network pharmacology, molecular docking facilitates the discovery of novel therapeutic compounds, enables the molecular-level prediction of ligand–target interactions, and provides a framework for deciphering structure–activity relationships [55]. A number of studies have employed molecular docking to explore cross-targets and related pathways of TPL across different diseases [56]. Common targets of TPL identified in multiple studies include AKT1, TP53, CASP3, TNF and STAT3 [57,58,59,60]. In addition, the NF-kB pathway has been reported as a key TPL-regulated pathway [59,61,62].

## 5. TPL Derivations and Delivery System

### 5.1. Chemical Structure of TPL’s Derivation

Due to its significant systemic toxicity (described below) and poor aqueous solubility, numerous derivatives have been synthesized to overcome these limitations. To the best of our knowledge, at least 20 TPL derivatives with confirmed in vitro cytotoxic effects have been reported (Table 2). The paramount common advantages of these derivatives are reduced toxicity and improved solubility [63]. Beyond these shared benefits, several derivatives exhibit specific mechanistic advantages: TRC102 potently reduces tRXRα expression and inactivates AKT [64]; LLDT-8 and LLDT-67 demonstrate neuroprotective effects against ischemic injury and Parkinson’s disease, respectively [65,66]; and MRx102 downregulates XIAP and Mcl-1, inhibits RNA transcription, and suppresses the Wnt signaling pathway [67].

### 5.2. TPL Delivery

A 2019 review comprehensively summarized TPL delivery systems into five types: nanoparticle encapsulation, oligonucleotide, peptide, sugar, and antibody conjugates [68]. Herein, we review the progress achieved from 2019 (summarized in Table 2).

The field of nanomaterials has witnessed explosive growth in recent years, attracting extensive research interest. Exosomes, as endogenous delivery systems that have gained prominence in recent years, exhibit targeted effects, reduced toxicity, and immune evasion capabilities. Liu et al. were the first to construct a TPL-loaded exosome delivery system, demonstrating superior antitumor efficacy compared with free TPL, along with reduced liver and spleen toxicity [69]. Another report combined exosomes and liposomes with oligonucleotides to co-deliver miR-497 and TPL, overcoming cisplatin resistance in ovarian cancer by activating the PI3K/AKT/mTOR pathway [70]. Similarly, Gu et al. developed hybrid nanoparticles encapsulating exosomes, liposomes, and CYP3A4-siRNA, which effectively inhibited melanoma growth with negligible toxicity in a mouse model [71].

Recently, biomimetic nanoparticles have shown considerable potential in prolonging circulation time, enhancing membrane penetration, and improving the solubility and stability of loaded drugs. Li et al. developed cancer cell membrane-camouflaged biomimetic Poly(lactic-co-glycolic acid) (PLGA) nanoparticles loaded with TPL for the treatment of hepatocellular carcinoma (HCC), which promoted tumor-site accumulation and reduced TPL toxicity [72]. Another study utilized a cancer cell–platelet hybrid membrane to co-deliver sorafenib and TPL, leveraging the advantages of both long circulation and homologous targeting [73]. Metal–organic frameworks (MOFs) have recently emerged as promising drug-delivery platforms due to their tunable pore sizes, large surface areas, and ease of functionalization. In one study, a TPL-loaded MOF coated with methotrexate enabled effective tumor accumulation and deep penetration, thereby remodeling the tumor microenvironment in triple-negative breast cancer [74].

**Table 2:** The derivation and delivery system for TPL.

Name	Molecular Formula *	Advantage	Reference
TPL	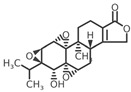		[16]
ZT01	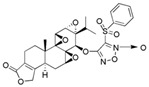	Strong anti-inflammatory effects and low toxicity; obviously beneficial effect on DSS-induced colitis	[75]
PG490-88 (Omtriptolide)	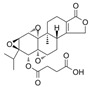	Highly effective in prevention of murine GVHD via inhibition of alloreactive T cell expansion through interleukin-2 production	[76]
LLDT-8	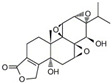	Inhibits T cell activation; reduces toxicity	[77]
LLDT-246	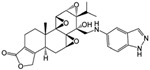	Suppresses NF-κB signaling by interpreting AKT/GSK3β/mTOR pathway on HCT-116 cells	[78]
LLDT-288	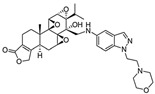	Efficacy in human prostate xenograft mice model with obviously low toxicity; no inhibitory effects on CYP450 isoforms	[79]
LLDT-67	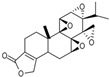	Neuroprotective effect: enhances NGF synthesis in astrocytes in the midbrain and rescue dopaminergic neurons indirectly through TrkA activation	[65]
Epoxide-transposition analogues of triptolide	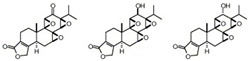	Cytotoxicity to A549+HT29	[80]
MRx-102	Not available	Decreased leukemia burden and increased survival time in mouse; Inhibited Wnt pathway in lung cancer	[67,81]
TP-disulfide-CR7 (TP-S-S-CR7)	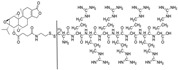	Reduce toxicity to skin and organ; No effect on the intracellular ROS;	[82]
Triptolidyl 2-(1-methylpiperidine-1-yl) acetate and a series of C-14 triptolide derivatives (17 types)	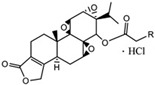	Decreases toxicity and increases water solubility; efficacy on imatinib-resistant CML	[83]
TRC4	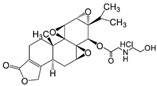	Decreases the nuclear retinoid X receptor-α; inactivates AKT and induces apoptosis	[64]
TPL-memantine	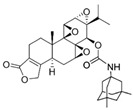	Neuroprotective effect against Aβ1–42 toxicity; inhibitory effect against LPS-induced TNF-α production	[84]
Minnelide	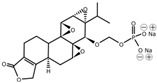	Increases water solubility and bioavailability; reduces systemic toxicity; clinical trial for leukemia, pancreatic and gastric cancer	[2,85]
Tryptophan (Trp), Valine (Val), and Lysine (Lys) conjugated to TPL	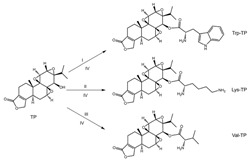	Pancreatic-cancer-selective delivery system; increases cytotoxicity	[86]
Triptolide aminoglycoside (TPAG)	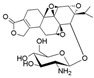	Increases kidney-targeting efficiency; protective effect against renal ischemia/reperfusion injury; low systemic toxicity	[87]
TP-CSO	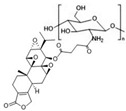	Increases water solubility; reduces systemic toxicity; increases half-life in blood circulation	[3]
Cet-TPL	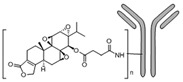	Target-specific cytotoxicity against EGFR-expressing cancer cells; reduced in vivo toxicity	[88]
CCTP	Not available	Reduced in vivo toxicity	[89]
CK21	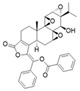	Inhibits NF-kB pathway; increases intracellular ROS; reduces toxicity in vivo	[90]
CL20	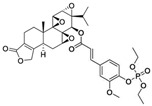	Strong cytotoxicity to human hepatoma	[91]
AS1411-triptolide conjugate (AS-TP)	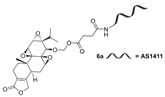	In situ triptolide release and increases intra-tumor triptolide accumulation; increases anti-TNBC efficacy and reduces in vivo toxicity	[92]
TPL loaded nanoparticle platform composed of L-ascorbate palmitate	Not available	Increases water solubility; reduces systemic toxicity; inhibits the erosion of synovitis and bone tissue	[93]
TP-PEG-SS assembled with ginsenoside Rg3 and lecithin to form nanovesicles	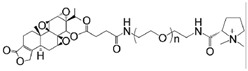	Targets mitochondria and M2 macrophage; selectively accumulates in the tumor; improves the immunosuppressive tumor microenvironment	[94]
Functionally modified triptolide liposome (FA+TPP-TP-Lips)	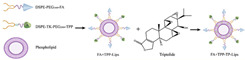	Accumulates in tumor tissues; improves their targeted delivery to mitochondria; reduces systemic toxicity	[95]
TP-P1	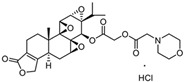	Improves water solubility and rapid release; inhibits acute myeloid leukemia in vivo; enhances the efficacy of FLT3 inhibitors	[96]
TPDMSA	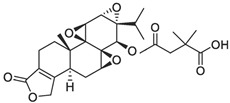	Suppresses influenza A virus replication and regulates innate immune response	[97]
TPL@TFBF	Not available	Triggers systemic antitumor immune responses; induces ferroptosis and pyroptosis; synthetic effects when combined with immune checkpoint blockade	[98]
A10 (one silyl ether-based linker conjugated with antibody drug)	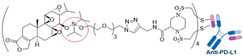	Targeted cytotoxicity for cells with high PD-L1 expression; bystander killing effect on cells with low PD-L1 expression; accumulates in tumor tissues	[99]
C60-SMEDDS/TP	Not available	Reduces toxicity to normal tissues	[100]
Na_2_GA&TP-BM	Not available	Increases cytotoxicity to tumor cells; increases water solubility; extends the blood circulation time with less system toxicity	[101]
TP-siRC@tHyNPs	Not available	Enhances targeted delivery through DR5 receptor; prolongs the half-life of TP and decreases its in vivo toxicity	[71]
Triptolidiol	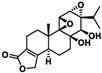	NLRP3 inhibitor; regulates inflammasome assembly and activation by decreasing K63-linked ubiquitination	[102]
TP-DEA2	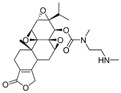	Improves water solubility; reduces toxicity; inhibits pulmonary fibrosis by reducing the secretion of a-SMA in fibroblasts	[103]
A9 (TPO–furoxan conjugation)	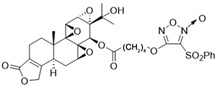	Enhances water solubility and safety; integrates NO-mediated ROS induction and FOCM inhibition	[104]
TP-PSP	Not available	Kidney-targeted delivery; enhances water solubility and reduces renal, cardiac, gastrointestinal, and hepatic toxicity	[105]
STP1	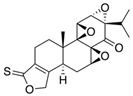	Modulates the differentiation of B cells into plasma cells and T cells into Tfh cells; regulates B-cell receptor and T-cell receptor signaling by directly targeting Fyn kinase	[106]

* The molecular formula were adopted from the corresponding references.

## 6. The Antitumor Mechanism and Preclinical Studies of TPL

### 6.1. XPB and RPB1 Are the Major Targets of TPL

**Figure 3:**
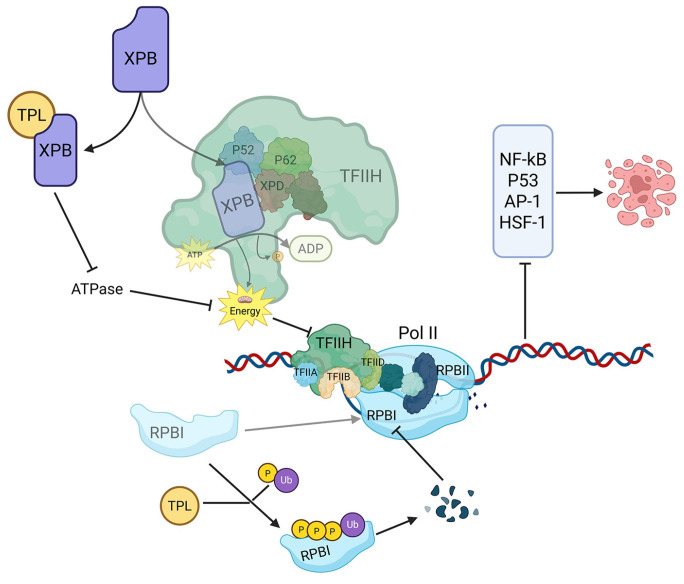
Mechanism of TPL inducing cell death. First, TPL covalently binds to the XPB subunit and inhibits its ATPase activity, thereby impairing the function of the TFIIH complex; Second, TPL promotes the phosphorylation and ubiquitination of RPB1, leading to its depletion. Together, these mechanisms inhibit the activity of key transcription factors, resulting in suppressed cell proliferation and induction of apoptosis (created with BioRender.com).

Multiple studies have shown that TPL inhibits de novo RNA synthesis, suggesting that RNA polymerases might be the target. However, the precise mechanisms were only elucidated recently (Figure 3). Titov et al. demonstrated that TPL covalently binds to XPB, a subunit of transcription factor TFIIH, thereby inhibiting its DNA-dependent ATPase activity [5]. In parallel, TPL induces rapid depletion of RPB1, the main subunit of RNA polymerase II, which is a hallmark of transcription elongation blockage. This is accompanied by Ser-5 hyperphosphorylation and increased ubiquitination within the RPB1 C-terminal domain [107,108]. Together, these two mechanisms inhibit the transcriptional activation of several transcription factors, including NF-κB, AP-1, p53, and HSF-1, ultimately leading to apoptosis and cell death.

### 6.2. Glioma

Glioma is a common intracranial malignant tumor characterized by high incidence, rapid progression, frequent recurrence, and poor prognosis. Over the past two decades, TPL has demonstrated promising potential in preclinical glioma models in vitro [109]. Subsequent studies have shown that TPL induces glioma cell apoptosis by modulating the NF-κB signaling pathway and promoting ROS generation [61]. In IDH1-mutant supratentorial gliomas, TPL disrupts glutathione metabolism, establishing a synthetic lethality with ROS [110]. Furthermore, TPL counteracts the immunosuppressive tumor microenvironment by reversing glioma-mediated inhibition of CD4+ T cells and promoting IFN-γ secretion, highlighting its immunomodulatory function [111].

TPL also enhances the radiosensitivity of glioma cells in vitro, suggesting a potential role as a radiosensitizer for high-grade gliomas [112]. When injected, TPL-preloaded hydrogel applied to the resected glioblastoma cavity demonstrated marked antitumor efficacy via ferroptosis and prolonged survival in an orthotopic relapse model [113]. Similarly, a dendrimer–TPL conjugate designed to target tumor-associated macrophages has also been shown to reduce tumor burden with minimal systemic exposure [114]. MicroRNA let-7b-5p has been identified as an important mediator of TPL’s anti-glioma activity [115].

### 6.3. Pancreatic Tumor

Pancreatic cancer, a highly aggressive malignancy, remains a paramount challenge in oncology. Current chemotherapeutic options are limited by their efficacy and selectivity. Recent efforts have focused on developing TPL-based prodrugs to improve TPL’s therapeutic profile. Wang et al. developed antibody–drug conjugates incorporating TPL via silyl ether linkers, which enhanced tumor-targeted cytotoxicity and demonstrated potent bystander-killing effects [99]. In a multifaceted approach, TPL prodrug nanovesicles co-loaded with ginsenoside Rg3 were designed to simultaneously target tumor mitochondria and reprogram immunosuppressive M2 macrophages, thereby remodeling the tumor microenvironment and reducing tumor burden in vivo [94]. Similarly, *Lycium barbarum* polysaccharide-modified selenium nanoparticles encapsulating TPL were shown to reduce systemic toxicity and enhance solubility [116]. To improve water solubility and therapeutic efficacy, Su et al. conjugated TPL to octreotide using a linker derived from succinic anhydride [117]. Beyond delivery systems, a novel TPL analog was designed to inhibit the NF-κB pathway, increase oxidative phosphorylation, and induce mitochondrial-mediated apoptosis [90]. Moser et al. identified TPL as a covalent inhibitor of XPB, demonstrating that it disrupts the TFIIH complex, induces RPB1 degradation, and synergizes with TRAIL to promote apoptosis [118]. TPL and its prodrug Minnelide exert antitumor effects by targeting the cell cycle, super-enhancers, the SP1 transcription factor, and the RAS signaling pathway in pancreatic in vivo models [119,120,121,122].

### 6.4. Leukemia

Leukemia comprises a heterogeneous group of hematological malignancies driven by genetic and epigenetic dysregulation. As leukemic cells are systemically distributed, the development of targeted delivery systems for TPL has received comparatively less attention than in solid tumors. Nevertheless, Kang et al. synthesized a series of water-soluble TPL prodrugs that exhibited faster and more complete release profiles than Minnelide and effectively suppressed leukemia growth in vivo [96]. Most research has centered on combination therapies and overcoming chemoresistance. TPL has been shown to potentiate the efficacy of various agents, including the BET inhibitor JQ1, the Bcl-2 inhibitor ABT-199, the XPO1 inhibitor selinexor, idarubicin, and Ara-C, through distinct synergistic pathways [4,123,124,125,126]. Furthermore, TPL can reverse chemoresistance, notably to adriamycin, by promoting ROS generation and disrupting the DNA damage response [126,127,128]. Apoptosis is a major antitumor mechanism of TPL in leukemia. Multiple signaling pathways and mechanisms mediate TPL’s proapoptotic effect. TPL induced apoptosis in leukemia cells by activating ROCK1 and phosphorylating MLC and MYPT1. Minnelide has also been extensively investigated. It induces apoptosis and cell cycle arrest by targeting the Ars2/miR-190a-3p/Akt pathway and downregulating the transcriptional regulator c-Myc, thereby inhibiting the growth of patient-derived leukemia cells in both in vitro and in vivo models [129,130].

### 6.5. Lung Cancer

Non-small-cell lung cancer (NSCLC), the most prevalent form of lung cancer, has been the subject of substantial research interest regarding the therapeutic potential of TPL. Initial investigations date back to 2002, when Lee et al. demonstrated that TPL sensitizes NSCLC cells to TRAIL-induced apoptosis by inhibiting NF-κB activation [131]. Subsequent studies have elucidated multiple molecular targets of TPL. It induced apoptosis and exerted antimetastatic effects by targeting the MAPK-ERK and MAPK-MKP pathways [132,133]. Furthermore, TPL activates ERK1/2 to stabilize p53, which in turn inhibits IκBα phosphorylation and NF-κB nuclear translocation, thereby blocking NF-κB-mediated survival signaling in NSCLC cells [134]. This downregulation of NF-κB can reverse paclitaxel resistance, a key mechanism of treatment failure [135]. Mechanistically, TPL also inhibits the PI3K/AKT pathway by reducing the expression of PFKFB2, a critical glycolytic enzyme required for cell growth [136,137]. Additionally, TPL directly binds to HNF1A, thereby attenuating the Sonic Hedgehog pathway and overcoming paclitaxel resistance [138]. From an immunotherapeutic perspective, TPL downregulates PD-L1 expression on NSCLC cells by suppressing the IFN-γ-JAK-STAT signaling axis, suggesting a potential role in modulating immune checkpoint inhibition [139]. It is noteworthy that, to the best of our knowledge, no studies have yet been published on the efficacy of TPL against small-cell lung cancer.

### 6.6. Other Cancers

TPL has demonstrated efficacy against a broad spectrum of other cancers in vitro and in vivo, often through shared pathways. For instance, a C60-modified self-micro-emulsifying drug-delivery system for TPL exhibited reduced cytotoxicity against normal cells compared with liver and gastric cancer cells in vitro [100]. Similarly, various TPL-loaded nanoplatforms have been developed for esophageal, hepatocellular, breast, and gastric cancers, demonstrating improved biosafety and enhanced on-target efficacy [140,141,142,143]. Mechanistically, TPL induces gastric cancer cell apoptosis by covalently binding to PRDX2, thereby elevating intracellular ROS levels [144]. In the context of cancer immunotherapy, TPL downregulates PD-L1 expression and suppresses the IFN-γ-mediated JAK2-STAT1 pathway in oral cancer [145]. Furthermore, TPL modulates different forms of cell death and stress responses. It induces cuproptosis, a novel copper-dependent cell death linked to metabolism, in cervical cancer by regulating the XIAP/COMMD1/ATP7A/B axis [146].

## 7. Clinical Trial

A systematic search of clinical trial registries, including the NIH clinical trial (https://clinicaltrials.gov), the European clinical trial (https://euclinicaltrials.eu/, accessed on 17 November 2025), and the Chinese clinical trial registry (https://www.chictr.org.cn/index.html, accessed on 17 November 2025), identified nine registered clinical trials investigating TPL (specifically its prodrug, Minnelide) for oncological indications, all listed on the NIH platform (Table 3). Among these, four trials have been completed, half of which were Phase I studies. Two Phase I trials conducted by the same research group demonstrated the safety and preliminary efficacy of Minnelide in patients with advanced gastrointestinal cancers [147,148]. One Phase II trial (NCT03117920) focusing on refractory pancreatic cancer was completed in 2023, but no results have been reported in the registry or in peer-reviewed publications. Another Phase II trial (NCT04896073) reported its outcomes: of the 16 enrolled patients, 12 completed the study. Grade 4 adverse events, primarily hematological (e.g., anemia, thrombocytopenia, leukopenia), occurred in 8.7–25% of patients, with no grade 5 events reported. Critically, no objective responses (complete or partial) were observed, and the median overall survival was 4.91 months (95% CI: 1.96–7.85). Currently, three Phase I trials for advanced NSCLC, pancreatic cancer, and solid tumors are in the recruitment phase, while one trial for acute myeloid leukemia was terminated due to dose-limiting toxicities (NCT03760523). Although preclinical studies continue to support Minnelide’s potential in other malignancies [2,85,130], its clinical translation requires further optimization. Notably, a growing body of research into novel TPL conjugates and delivery systems has shown superior preclinical profiles (Table 2), suggesting promising alternatives for future clinical development.

**Table 3:** The clinical trials associated with TPL in oncology.

ID	Title	Tumor Type	Phase	Intervention	Status	Conclusion	Reference *
NCT04896073	Superenhancer Inhibitor Minnelide in Advanced Refractory Adenosquamous Carcinoma of the Pancreas	Advanced Refractory Adenosquamous Carcinoma of the Pancreas	II	Minnelide	Completed	Platform data without publication. A total of 16 patients were enrolled and 12 patients completed the trial. The investigator provided baseline characteristics including age, sex, ethnicity, race, and region. In term of side effects, 8.7–25% patients showed grade 4 side effects, mainly related to blood cell (anemia, platelet and white blood cell) count decrease, without grade 5 side effects. However, most importantly, neither patient showed complete response nor partial response. Overall survival found to be 4.91 (1.96–7.85) months.	[149]
NCT03117920	A Phase II, International Open Label Trial of Minnelide in Patients with Refractory Pancreatic Cancer	Refractory Pancreatic Cancer	II	Minnelide	Completed	No results posted on the platform or publication.	ClinicalTrials.gov (NCT03117920)
NCT05566834	Minnelide Capsules Alone and in Combination with Paclitaxel in Advanced Gastric Cancer (AGC)	Advanced Gastric Cancer	I	Minnelide	Completed	Minnelide alone at a dose of 1.25 mg was tolerable for AGC patients and the combination of Minnelide and paclitaxel exhibited meaningful clinical efficacy alongside a manageable safety profile.	[147]
NCT01927965	Study of Minnelide in Patients with Advanced GI Tumors	Advanced Gastrointestinal Carcinoma	I	Minnelide	Completed	The trial identified a dose and schedule of Minnelide in patients with refractory GI cancers and observed efficacy of Minnelide treatment. Grade ≥ 3 toxicities occurred in 69% of patients; the most common side effect was neutropenia (38%).	[148]
NCT05166616	Minnelide and Osimertinib for the Treatment of Advanced EGFR Mutated Non-small-cell Lung Cancer	Advanced EGFR Mutated NSCLC	Ib	Minnelide + osimertinib	Recruiting		ClinicalTrials.gov (NCT05166616)
NCT03129139	A Phase 1, Multi-Center, Open-Label, Dose-Escalation, Safety, Pharmacokinetic, and Pharmacodynamic Study of Minnelide Capsules Given Alone or in Combination with Protein-Bound Paclitaxel in Patients With Advanced Solid Tumors	Advanced Solid Tumors	I	Minnelide	Recruiting		ClinicalTri-als.gov (NCT03129139)
NCT05557851	Minnelide Along with Abraxane Plus Gemcitabine in Patients With Metastatic Adenocarcinoma of the Pancreas	Metastatic Adenocarcinoma of the Pancreas	Ib	Minnelide + Abraxane + gemcitabine	Recruiting		ClinicalTri-als.gov (NCT05557851)
NCT03760523	Dose Escalation Study of Minnelide in Relapsed or Refractory Acute Myeloid Leukemia	Relapsed or Refractory Acute Myeloid Leukemia	I	Minnelide	Terminated	Two dose-limiting toxicity events occurred.	ClinicalTri-als.gov (NCT03760523)
NCT03347994	Minnelide in Adult Patients with Relapsed or Refractory Acute Myeloid Leukemia (AML)	Relapsed or Refractory AML	I	Minnelide	Withdrawn	Discordance in contractual language and terms.	ClinicalTri-als.gov (NCT03347994)

* Completed trials with published results are cited by reference numbers; ongoing or completed trials without published results are referenced by their ClinicalTrials.gov identifier.

## 8. Conclusions

Despite its broad biological activities, including antitumor, anti-inflammatory, immunosuppressive, and neuroprotective effects, the clinical application of TPL is significantly limited by its systemic toxicity [150,151,152]. To overcome this challenge, numerous derivatives and drug-delivery systems have been developed to mitigate toxicity and enhance on-target efficacy. Among them, Minnelide, a water-soluble prodrug derived from TPL by adding a phosphate group, has emerged as one of the most promising candidates and has been widely evaluated in clinical trials owing to its reduced toxicity [85]. According to major clinical trial registries, Minnelide has been involved in nine trials across several malignancies, including leukemia, pancreatic, gastric, and lung cancers. Importantly, Minnelide has demonstrated an acceptable safety profile in patients, with the main adverse event being reversible acute cerebellar toxicity. However, since Minnelide is reconverted to TPL by phosphatases in vivo, it does not fully resolve either the compound’s inherent toxicity or its on-target effect [85]. Consequently, alternative delivery strategies using novel materials, including MOF, PLGA nanoparticles, and cancer cell–platelet hybrid membranes, have been explored [72,73,74]. Nevertheless, their clinical translation remains hampered by challenges in production costs, quality control, biocompatibility, and potential long-term toxicity.

Alternatively, combination therapy is a viable strategy for reducing the required doses of individual drugs, targeting multiple mechanisms, delaying the development of resistance, and improving therapeutic efficacy [153]. Given the systemic toxicity observed in DMG preclinical models, alternative combination therapies remain a promising approach to enhance the clinical profile of TPL. Such prospective combinations may provide the opportunity to reduce TPL toxicity by using a lower dose of the drug.

## Data Availability

No new data were created or analyzed in this study. Data sharing is not applicable to our review.

## Abbreviations

The following abbreviations are used in this manuscript:

TPL	triptolide
ROS	reactive oxygen species
GPX4	glutathione peroxidase 4
PK	pharmacokinetic
DMG	diffuse midline glioma
Cmax	maximum concentration
Tmax	time to maximum concentration
*TwHF*	*Tripterygium wilfordii Hook. f.*
HCC	hepatocellular carcinoma
MOFs	metal–organic frameworks
NSCLC	non-small-cell lung cancer
PLGA	Poly (lactic-co-glycolic acid)
TF	transcription factor
AGC	advanced gastric cancer
AML	acute myeloid leukemia
